# Fast and efficient electrocatalytic oxidation of glucose triggered by Cu_2_O-CuO nanoparticles supported on carbon nanotubes

**DOI:** 10.3389/fchem.2022.998812

**Published:** 2022-09-01

**Authors:** Zhongting Wang, Yi Liu, Yongxi Cheng, Yu-Long Men, Peng Liu, Lei Zhang, Bin Dai, Yun-Xiang Pan

**Affiliations:** ^1^ School of Electronic Information and Electrical Engineering, Shanghai Jiao Tong University, Shanghai, China; ^2^ Department of Chemical Engineering, School of Chemistry and Chemical Engineering, Shanghai Jiao Tong University, Shanghai, China; ^3^ Beijing Institute of Aerospace Testing Technology, Beijing, China; ^4^ Department of Vascular Surgery, Changhai Hospital, Naval Medical University, Shanghai, China

**Keywords:** copper oxide, glucose oxidation, synergistic effect, electrocatalysis, portable detection

## Abstract

Electrocatalytic glucose oxidation reaction (GOR) is the key to construct sophisticated devices for fast and accurately detecting trace glucose in blood and food. Herein, a noble-metal-free Cu/C-60 catalyst is fabricated by supporting Cu_2_O-CuO nanoparticles on carbon nanotubes through a novel discharge process. For GOR, Cu/C-60 shows a sensitivity as high as 532 μA mM^−1^ cm^−2^, a detection limit as low as 1 μM and a steady-state response time of only 5.5 s. Moreover, Cu/C-60 has outstanding stability and anti-interference ability to impurities. The synergistic effect of Cu_2_O-CuO could improve the adsorption and conversion of glucose, thus enhancing GOR performance. By using Cu/C-60, we fabricate a three-electrode chip. A portable and compact electrochemical system is constructed by connecting the three-electrode chip with Cu/C-60 to an integrated circuit board and a mobile phone for recording and displaying data. The portable and compact electrochemical system results in a GOR sensitivity of 501 μA mM^−1^ cm^−2^, which is close to the data measured on the bloated electrochemical workstation. The detection limit of the portable and compact electrochemical system in GOR is 50 μM. This is higher than those obtained on the bloated electrochemical workstation, but is much lower than the common blood glucose concentration of human body (>3 mM). This demonstrates the accuracy, reasonability and applicability of the portable and compact electrochemical system. The results of the present work are helpful for fabricating fast, efficient and portable devices for detecting trace amount of glucose in blood and food.

## Introduction

Electrocatalytic glucose oxidation reaction (GOR) is the key to construct sophisticated devices for fast and accurately detecting the trace amount of glucose in blood and food in areas of life, biomedicine and food (Galant et al., 2015; Liu et al., 2020; Peng et al., 2020; Dong et al., 2021; Jiang et al., 2021; Parrilla and De Wael, 2021). A crucial factor determining the devices’ efficiency in detecting glucose is the GOR catalysts. The GOR catalysts are usually evaluated by sensitivity (the current density induced by the catalysts toward 1 mM glucose in 1 cm^−2^ electrode), detection limit (the lowest glucose amount that be able to be detected by the catalysts, μM), response time (the time that the catalysts use for responding to a certain amount of glucose) and anti-interference ability (the ability of the catalysts to resist the influence of impurities) (Peng et al., 2020; Dong et al., 2021; Jiang et al., 2021). The GOR catalysts with higher sensitivity, lower detection limit, shorter response time and higher anti-interference ability are highly desired for fabricating more fast and accurate glucose detection devices.

The most widely used GOR system loaded glucose oxidase or glucose dehydrogenase on noble metal electrode, and applied enzyme to provide catalytic active sites for GOR (Yoon et al., 2020; Lu et al., 2021). However, the enzyme is sensitive to temperature, pH and impurities, and the structures of enzyme can be easily destroyed by high temperature, strong acid, strong alkali and impurities, thus making the enzyme-based catalytic reaction system poor in stability, reusability and anti-interference to impurities. For solving these problems, many noble-metal-based enzyme-free GOR catalysts have been developed (Zhu et al., 2012; Liu et al., 2013; Lee et al., 2018; Shim et al., 2019). For example, Lee et al*.* (2018) reported a nanoporous Pt catalyst with an enhanced GOR sensitivity, and Zhu et al. (2012) found that the bimetallic Pd-Au alloy nanowire catalyst had a GOR sensitivity of 533 μA mM^−1^ cm^−2^ which was higher than that obtained when using Au and Pd catalysts separately. Despite the extensive studies, the noble-metal-based enzyme-free GOR catalysts have poor anti-interference to impurities, e.g., NaCl, which are commonly present along with glucose in blood and food, and can be easily oxidized or poisoned by oxidants formed in the GOR process (e.g., OH groups). These lead the noble-metal-based enzyme-free GOR catalysts to be poor in stability and reusability. In addition, the scarce resources and high price of noble metals make the noble-metal-based enzyme-free GOR catalysts be unsuitable for large-scale commercialization.

Low-cost noble-metal-free catalysts without enzyme, e.g., Cu-based catalysts, have attracted great attentions for GOR (Shu et al., 2017; Barragan et al., 2018; Ling et al., 2018; Dourado et al., 2019; Li et al., 2019; Zhou et al., 2020; Ahmad et al., 2021; Pathak et al., 2021; Xu et al., 2021; Men et al., 2022a). Shu et al*.* (2017) found that the synergistic effect between Ni nanoparticles and NiO nanoparticles decorated on metal-organic frameworks (MOF) nanosheets led to a detection limit of 0.8 μM and a sensitivity of 367.45 μA mM^−1^ cm^−2^ in GOR. Xu et al. (2021) observed a GOR sensitivity as high as 3250 μA mM^−1^ cm^−2^ on Ni/Co decorated MOF. They proposed that the synergistic effect between Ni and Co facilitated the electron transfer among Ni, Co, and MOF, thus enhancing the GOR sensitivity (Xu et al., 2021). Doping Ni atoms into Co_3_O_4_ to form NiCo_2_O_4_ has been demonstrated to significantly increase the conductivity, surface area and amount of the active sites for GOR, and thereby resulted in a higher GOR sensitivity as compared with that obtained by using Co_3_O_4_ (Pathak et al., 2021). Ling et al*.* (2018) fabricated a Cu@Cu_2_O aerogel network catalyst for GOR. The Cu@Cu_2_O aerogel network catalyst exhibited a detection limit of 15.0 μM, which was lower than that of Cu_2_O nanocubes (38 μM) and that obtained by growing Cu_2_O directly on Cu (37.0 μM) (Ling et al., 2018). In addition, a good linear relationship was observed for glucose concentrations ranging from 50.0 to 8.0 mM at a relative low poised potential of 0.55 V during the GOR on the Cu@Cu_2_O aerogel network catalyst (Ling et al., 2018). It was proposed that the redox couple of CuO/CuOOH and the cross-linking structure of Cu and Cu_2_O on the Cu@Cu_2_O aerogel network catalyst could be the origins for the enhanced GOR performance (Ling et al., 2018). In spite of extensive studies, the efficiency of noble-metal-free catalysts without enzyme in GOR is still far below the need of commercialization, and requires further improvement.

Herein, we support copper oxide nanoparticles containing Cu_2_O and CuO on carbon nanotubes (CNTs) through a novel discharge process to fabricate a noble-metal-free catalyst without enzyme, denoted by Cu/C-60 for clarity. The discharge process has been detailed described in our previous work (Men et al., 2022a). It is formed by ionizing Ar by a voltage of 100 V at about 150°C. Only Ar is used as the working gas during the discharge process, without using any harmful substance and without heating. Abundant high-energy electrons with energies of 5–10 eV are produced from the discharge process, and trigger the dissociation of metal salts into metal oxides. Cu/C-60 exhibits significantly improved activity, sensitivity, anti-interference ability and stability towards GOR. The synergistic effect between Cu_2_O and CuO as well as the CNTs support could be the origin for the improved GOR efficiency of Cu/C-60. By using Cu/C-60 as GOR catalyst, we construct a portable and compact mobile-phone readable glucose detector, and achieve a fast, convenient, and efficient detection on trace amount of glucose.

## Methods

### Material syntheses

Cu/C-60 was fabricated *via* the following procedures. Firstly, 1.0 g CNTs were uniformly dispersed in a Cu(CH_3_COO)_2_∙3H_2_O solution (0.3 M, 7.3 ml) under ultrasonication at room temperature to form a mixture. Secondly, the mixture from the first step was kept for 12 h for impregnating the copper ions on CNTs. Thirdly, the sample was dried at 110ºC for 12 h. Fourthly, the dried sample was calcined at 350°C for 2 h under Ar atmosphere, leading to the formation of a sample named as Cu/C-0 for clarity. Fifthly, the Cu/C-0 sample was separated into two parts. One part of Cu/C-0 was characterized and applied in GOR. The other part of Cu/C-0 was treated by using a dielectric barrier discharge process for 60 min, resulting in Cu/C-60. Supplementary Figure S1 schematically shows the set-up for the discharge process. During the discharge process, 500 mg Cu/C-0 was firstly put into the discharge chamber. Then, a voltage of 100 V and a current of 2A was applied on the electrodes of the discharge chamber to trigger the discharge process in the presence of air under atmospheric pressure (Men et al., 2022a). In the discharge process, abundant high-energy electrons with energies of 5–10 eV are produced from air ionization and move fast (Sabat et al., 2016; Dou et al., 2018; Men et al., 2020; Men et al., 2022a; Men et al., 2022b). Collisions of the high-energy electrons with the species in the discharge chamber can efficiently dissociate metal salts into metal oxides, reduce metal ions into metal atoms with valence in zero, control the transfer and aggregation of metal atoms to form highly dispersed metal nanoparticles with uniform sizes, and trigger various reactions among different species (Sabat et al., 2016; Dou et al., 2018; Men et al., 2020; Men et al., 2022a; Men et al., 2022b). For comparison, we also prepared Cu/C-10, Cu/C-20, Cu/C-30 and Cu/C-120 through treating Cu/C-0 by using the discharge process for 10, 20, 30, and 120 min, respectively.

### Material characterizations

X-ray diffraction (XRD) patterns were obtained on a Rigaku Ultimate IV powder X-ray radiation diffractometer (Cu Kα, 40 kV and 40 mA). X-ray photoelectron spectroscopy (XPS) analyses were conducted on a Thermo Scientific K-Alpha spectrometer using Al Kα X-ray source (1486.6 eV) operated at 12 kV. The binding energy was calibrated with the C 1s peak at 284.6 eV. Transmission electron microscopy (TEM) observations, including high resolution TEM (HRTEM) images, were conducted on a transmission electron microscope (JEOL JEM 2100F). Energy Dispersive Spectroscopy (EDS) images were obtained on a spectrometer (JED 2300). N_2_ adsorption-desorption measurements were done on a Quantachrome NOVA specific surface and pore size analyzer. Surface areas were calculated by the Brunauer-Emmett-Teller (BET) method. The contact angle data were measured on a Kruss DSA10 Droplet shape analyzer.

### Electrochemical measurements

All electrochemical measurements were performed on an electrochemical workstation (CHI760E, CH Instrument, Inc.) at room temperature with a three-electrode system in an aqueous solution containing 0.10 M NaOH. The working electrode was prepared by using a circular glassy carbon electrode (GCE) (*d* = 0.30 cm). 2 mg catalyst and 10 μl Nafion (5%) were added into 300 μl ethanol solution (65%), and a homogeneous ink was obtained after 5 min ultrasonication. 10 μl ink was dropped on the polished GCE surface, followed by drying at room temperature for 12 h, leading to the formation of the working electrode. A Pt plate electrode (0.50 cm × 0.50 cm) and a saturated calomel electrode (SCE) were used as the counter and reference electrodes, respectively. To achieve stable electrical signal acquisition, the electrodes were scanned by cyclic voltammetry (CV) for 50 cycles at a scan rate of 100 mV s^−1^ in the potential range of 0.00–0.80 V (vs. SCE) before all electrochemical testing. The measured potentials were converted uniformly according to Eq. 1:

$$E_{\left(RHE\right)}\;=\;E_{\left(SCE\right)}+\;0.2415\;+\;0.0591\;\times \text{ pH}$$ (1)

where *E*_*(SCE)*_ and *E*_*(RHE)*_ are the potential measured against SCE and calibrated versus reversible hydrogen electrode (RHE), respectively.

Performances of catalysts in electrocatalytic GOR were explored in an aqueous solution of NaOH (0.10 M, 50 ml). CV scans (0.00–0.80 V vs. SCE) at different glucose concentrations were performed by continuously adding a small amount of glucose (0.20 mM) into the reaction solution at regular intervals. The current densities as a function of time and as a function of concentration were recorded for studying the performance of catalysts in GOR. The electrochemical double-layer capacitance (*C*_*dl*_) was evaluated by CV measurements in a non-Faradaic potential range at scan rates from 40 to 160 mV s^−1^. To ensure that the reaction solution evenly dispersed, we added the glucose into the reaction solution under stirring with a speed of 200 rpm. After adding the glucose, the reaction solution was stirred for 5 min, and then the electrocatalytic measurements were conducted. To determine the influence of the potential on the measurements, the current densities as a function of time at different potentials were investigated (Supplementary Figure S2). It was found that a potential of 0.50 V (vs. SCE) can give stable and evident current densities. Thus, the 0.50 V (vs. SCE) potential was used for all measurements.

## Results and discussion

### Catalytic performances

**FIGURE 1:**
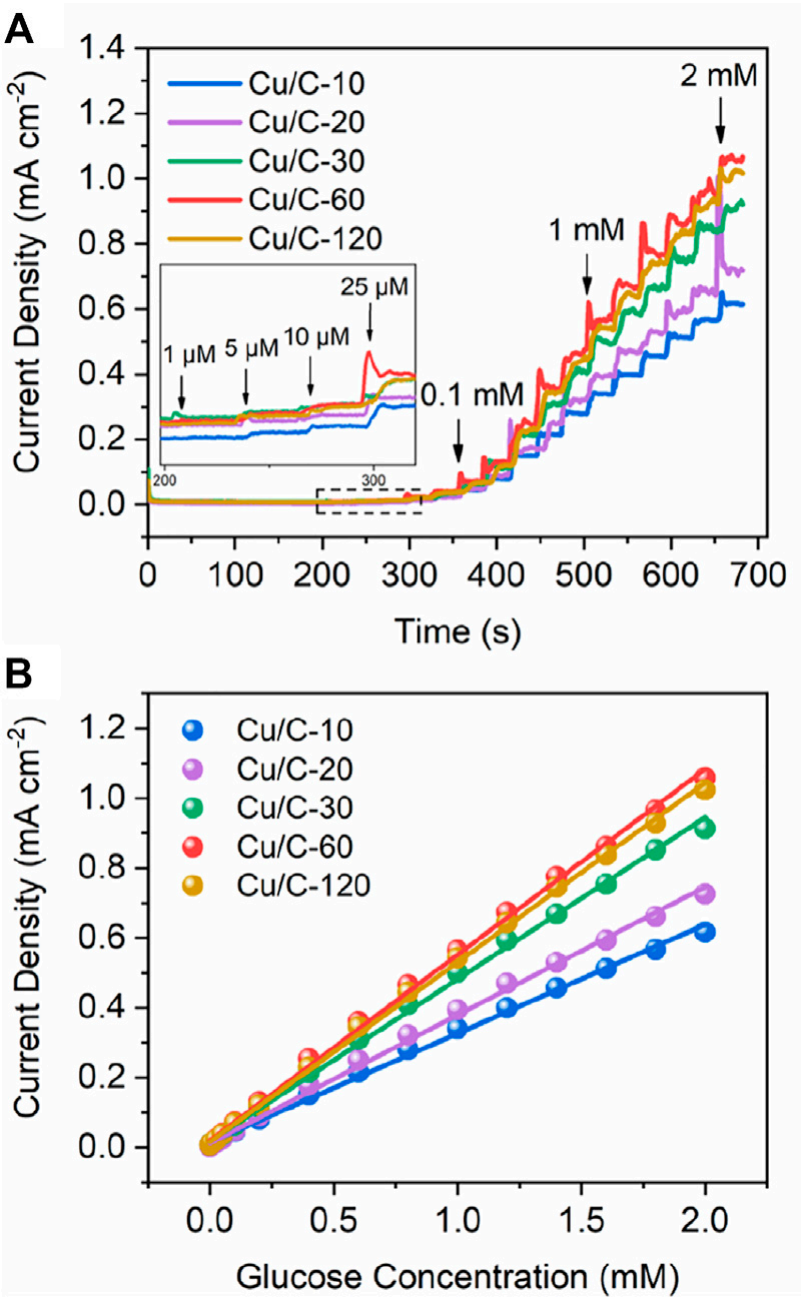
Current densities as functions of **(A)** time and **(B)** glucose concentration. The experiments are firstly conducted without glucose in the reaction solution from 0 to 210 s. At 210, 240, 270, 300, 330, 360, and 390s, 0.001, 0.005, 0.01, 0.025, 0.05, 0.10, and 0.20 mM glucose are added into the reaction solution separately. After 390 s, 0.20 mM glucose is added into the reaction solution for every 30 s. The glucose concentration shown in the figures are the total glucose concentration in the reaction solution.

To optimize the discharge time for preparing catalyst, we firstly explore the GOR performance of catalysts prepared by different discharge times. Figure 1A shows the current densities on Cu/C-10, Cu/C-20, Cu/C-30, Cu/C-60, and Cu/C-120 as a function of time. The current density at a certain glucose concentration reflects the GOR activity of catalyst, and a higher current density indicates a higher GOR activity. As implied in Figure 1A, along with elongating discharge time for preparing catalysts, the GOR activity increases firstly, and then decreases, with the highest GOR activity being achieved on Cu/C-60. Figure 1B illustrates the current densities on Cu/C-10, Cu/C-20, Cu/C-30, Cu/C-60, and Cu/C-120 as a function of glucose concentration. Calibration on the (current density)-(glucose concentration) relationship of the catalyst leads to a straight line, and the slope of the line reveals the GOR sensitivity of the catalyst, i.e., the current density induced by the catalyst to 1 mM glucose in 1 cm^−2^ electrode. According to Figure 1B, the GOR sensitivities of Cu/C-10, Cu/C-20, Cu/C-30, Cu/C-60 and Cu/C-120 are calculated to be 312, 367, 463, 532, and 514 μA mM^−1^ cm^−2^, respectively (Supplementary Figure S3). Thus, when the discharge time becomes longer, the GOR sensitivity increases firstly, and then decreases, with Cu/C-60 having the highest GOR sensitivity.

**FIGURE 2:**
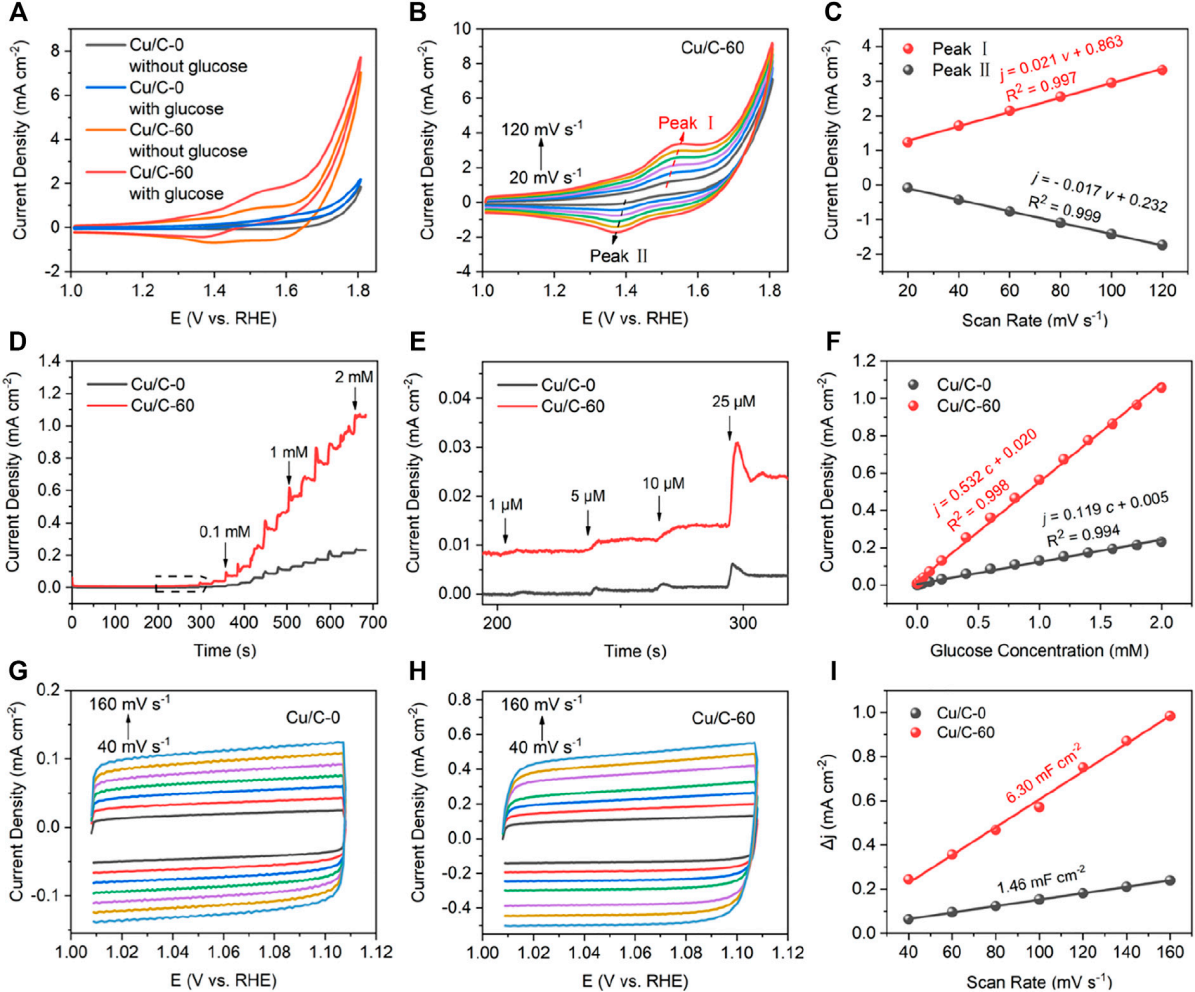
**(A)** CV curves obtained without glucose and with glucose (1 mM) at a scan rate of 40 mV s^−1^. **(B)** CV curves for Cu/C-60 obtained with 1 mM glucose at different scan rates. **(C)** Peak current densities of Peaks I and II in **(B)** as function of scan rates. **(D,E)** Current densities as function of times, with **(E)** being the enlarged drawing for the contents marked by dotted box in **(D)**. **(F)** Current densities as function of glucose concentrations. **(G,H)** CV curves in the non-Faradaic region at different scan rates, with **(G)** for Cu/C-0 and **(H)** for Cu/C-60. **(I)** Changes in current densities as function of scan rates in the non-Faradaic region. The glucose concentration shown in the figures are the total glucose concentration in the reaction solution.

We next compare the GOR performance of Cu/C-60 with that of Cu/C-0 prepared without using the discharge process (Figure 2). As reflected by the CV curves in Figure 2A, when adding 1 mM glucose into the reaction solution, Cu/C-60 shows a much lower initial potential for glucose oxidation, and results in a much evident glucose oxidation peak at about 1.53 V, as compared with Cu/C-0, indicating the much higher GOR activity of Cu/C-60 than that of Cu/C-0 (Barragan et al., 2018). The peaks at about 1.53 (Peak Ⅰ) and 1.37 V (Peak Ⅱ) on the CV curves of Cu/C-60 are assigned to the glucose oxidation and reduction from Cu^2+^ to Cu^+^, respectively, and become stronger with increasing scan rates from 20 to 120 mV s^−1^ (Figure 2B) (Gopiraman et al., 2013; Xiao et al., 2014; Peng et al., 2020). Based on the CV curves in Figure 2B, relationships of the peak current densities on Peaks Ⅰ and Ⅱ with the scan rates can be fitted into Eqs. 2,3, respectively (Figure 2C).

$$j\;=\;0.021\nu +0.863\left(R^{2}\;=\;0.997\right)$$ (2)

$$j\;=-0.017\nu +0.232\left(R^{2}\;=\;0.999\right)$$ (3)

where *j* and *ν* are current density and scan rate, respectively. The *j*-*ν* relationships for Peaks I and II are well fitted into straight lines (Figure 2C). This reveals that GOR on Cu/C-60 is a surface adsorption-controlled process, and has an excellent reversibility (Zhu et al., 2012; Xiao et al., 2014; Lee et al., 2018; Shim et al., 2019; Peng et al., 2020; Zhou et al., 2020).

Figures 2D,E plot current densities as function of times for Cu/C-0 and Cu/C-60. The experiments are firstly conducted without glucose in the reaction solution from 0 to 210 s. Then, 0.001, 0.005, 0.01, 0.025, 0.05, 0.01, and 0.20 mM glucose are added into the reaction solution at 210, 240, 270, 300, 330, 360, and 390 s, respectively. After 390 s, 0.20 mM glucose is added into the reaction solution for every 30 s. With increasing glucose concentration and time, both Cu/C-0 and Cu/C-60 exhibit a stepwise increase in current density. Even at a glucose concentration of 1 μM, the stepwise increase in current density on Cu/C-0 and Cu/C-60 are also evident, indicating that the detection limits for Cu/C-0 and Cu/C-60 reach to 1 μM. The stepwise increase in current density on Cu/C-60 is much more evident than that on Cu/C-0, indicating that Cu/C-60 has much higher GOR activity and sensitivity than Cu/C-0. In addition, the response time of Cu/C-60 to 1 μM glucose (5.5 s) is close to that of Cu/C-0 (5.6 s) (Supplementary Figure S4). With increasing the glucose concentration to 2 mM, the response time can be further decreased to 3.0 s (Supplementary Figure S4). Figure 2F plots the current densities on Cu/C-0 and Cu/C-60 as function of glucose concentrations. The (current density)-(glucose concentration) relationships for Cu/C-0 and Cu/C-60 can be fitted into straight lines described by Eqs. 4, 5, respectively.

$$j=0.119c+0.005\left(R^{2}=0.994\right)$$ (4)

$$j=0.532c+0.020\left(R^{2}=0.998\right)$$ (5)

where *c* is the glucose concentration. The slopes of the (current density)-(glucose concentration) lines for Cu/C-0 and Cu/C-60, i.e., GOR sensitivity, are calculated to be 119 and 532 μA mM^−1^ cm^−2^, respectively. Thus, the GOR sensitivity of Cu/C-60 is about 4.5 times higher than that of Cu/C-0. After proceeding GOR for 2500 s, the current density on Cu/C-60 is almost unchanged (Supplementary Figure S5). To measure the anti-interference ability of Cu/C-60, we continuously add KCl (0.1 mM), NaCl (0.1 mM), glucose (0.1 mM), ascorbic acid (AA, 0.1 mM), lactic acid (LA, 0.1 mM), uric acid (UA, 0.1 mM), urea (0.1 mM), and glucose (0.1 mM) into the reaction solution. Cu/C-60 shows fast and strong response to glucose, but no evident response to other species (Supplementary Figure S6), revealing the excellent anti-interference ability of Cu/C-60 to impurities.

CV curves in the non-Faradaic region at different scan rates are next explored to further compare the GOR performances of Cu/C-0 and Cu/C-60 by using double layer capacitance (*C*_*dl*_) and electrochemical active surface area (ECSA) (Yang et al., 2020; Liu et al., 2021). Figures 2G,H illustrates the CV curves at scan rates from 40 to 160 mV s^−1^ for Cu/C-0 and Cu/C-60, respectively. For calculating *C*_*dl*_, the current density difference ∆*j* is firstly obtained from Eq. 6:

$$\Delta j\;=\;j_{\mathrm{anodic}}\;-\;j_{\mathrm{cathodic}}$$ (6)

where *j*_anodic_ and *j*_cathodic_ are the anodic and cathodic current densities at 1.06 V on the CV curves, respectively. And then, ∆*j* is plotted as a function of scan rate in Figure 2I. The ∆*j*-(scan rate) relationships for Cu/C-0 and Cu/C-60 can be fitted into straight lines. *C*_*dl*_ is obtained by calculating the slope of the fitting line of the ∆*j*-(scan rate) relationship. The *C*_*dl*_ for Cu/C-60 (6.30 mF cm^−2^) is 4.3 times higher than that of Cu/C-0 (1.46 mF cm^−2^) (Figure 2I). ECSA is obtained from dividing *C*_*dl*_ by *C*_*s*_. *C*_*s*_ refers to the specific capacitance for an ideal flat surface of catalyst. 0.04 mF cm^−2^ has been widely used for *C*_*s*_ in alkaline solutions (Han et al., 2019). Larger *C*_*dl*_ and ECSA indicate a higher GOR activity. Therefore, Cu/C-60 has a higher GOR activity than Cu/C-0. In addition, by observing the CV curves in the non-Faradaic region at different scan rates (Supplementary Figure S7), the *C*_*dl*_ of Cu/C-10, Cu/C-20, Cu/C-30, and Cu/C-120 are calculated to be 3.78, 4.60, 5.65 and 5.97 mF cm^−2^, respectively (Supplementary Figure S8). Thus, Cu/C-60 has a higher GOR activity than other discharge-prepared catalysts.

### Catalyst characterizations

As discussed above, the GOR activity and sensitivity of Cu/C-0, Cu/C-10, Cu/C-20, Cu/C-30, Cu/C-60, and Cu/C-120 are in an order of Cu/C-0 < Cu/C-10 < Cu/C-20 < Cu/C-30 < Cu/C-60 > Cu/C-120, with the GOR activity and sensitivity of Cu/C-60 higher than other catalysts. We next characterize the catalysts for exploring the origin for the difference in GOR performance. N_2_ adsorption-desorption and contact angle measurements indicate that Cu/C-0, Cu/C-10, Cu/C-20, Cu/C-30, Cu/C-60, and Cu/C-120 have similar surface areas, porous properties and hydrophicilities (Supplementary Figure S9). Therefore, these factors could not be the origins for the higher GOR activity and sensitivity of Cu/C-60 than other catalysts.

**FIGURE 3:**
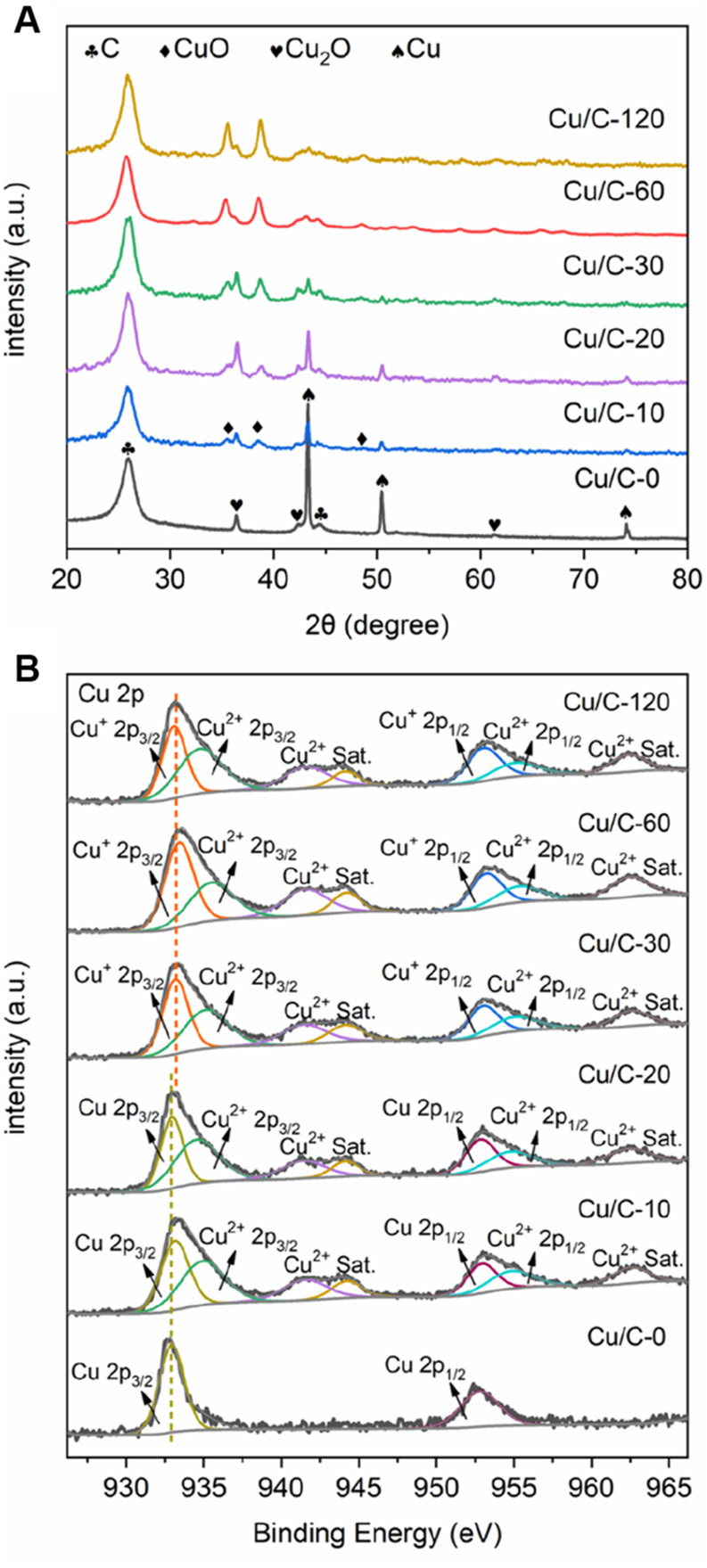
**(A)** XRD patterns and **(B)** Cu 2p XPS spectra for Cu/C-0, Cu/C-10, Cu/C-20, Cu/C-30, Cu/C-60 and Cu/C-120.

Figure 3A gives the XRD patterns of Cu/C-0, Cu/C-10, Cu/C-20, Cu/C-30, Cu/C-60 and Cu/C-120. The XRD peaks at 26.0 and 44.4º on all catalysts are caused by CNTs (Men et al., 2022a). In addition to the peaks of CNTs, the XRD pattern of Cu/C-0 also exhibits three strong peaks at 43.3, 50.4, and 74.1º as well as three weak peaks at 36.4, 42.4, and 61.4º. The strong peaks at 43.3, 50.4, and 74.1º are attributed to the (111), (200), and (220) planes of metallic Cu^0^ respectively (PDF#85–1326), while the weak peaks at 36.4, 42.4, and 61.4º are due to the (111), (200), and (220) planes of Cu_2_O respectively (PDF#75–1531). Thus, both metallic Cu^0^ and Cu_2_O exist on Cu/C-0 prepared without using the discharge process. The much stronger XRD peaks of metallic Cu^0^ than those of Cu_2_O indicates that metallic Cu^0^ could be dominate copper species on Cu/C-0, and the Cu_2_O amount on Cu/C-0 could be much smaller than that of metallic Cu^0^. In the calcination process for preparing Cu/C-0, the Cu^2+^ ions in Cu(CH_3_COO)_2_∙3H_2_O could be reduced into Cu^+^ of Cu_2_O and metallic Cu^0^ by the H atoms of functional groups like COOH or/and C atoms on CNTs (Gopiraman et al., 2013; Ai et al., 2017).

On the XRD patterns of Cu/C-10, Cu/C-20, and Cu/C-30, in addition to the peaks of CNTs, metallic Cu^0^ and Cu_2_O, three peaks at 35.3, 38.7, and 48.6 º are also present, and are assigned to the (200), (111), and (−202) planes of CuO, respectively (PDF#80–1917). Thus, the copper species on Cu/C-10, Cu/C-20, and Cu/C-30 include metallic Cu^0^, Cu_2_O, and CuO. The XRD patterns of Cu/C-60 and Cu/C-120 show peaks of Cu_2_O and CuO, without peaks of metallic Cu^0^, indicating that the copper species on Cu/C-60 and Cu/C-120 are mainly composed of Cu_2_O and CuO. The much stronger XRD peaks of CuO than those of Cu_2_O indicates that CuO could be dominate copper species on Cu/C-60 and Cu/C-120, and the Cu_2_O amount on Cu/C-60 and Cu/C-120 could be much smaller than that of CuO. As revealed in Figure 3A, along with increasing the discharge time for preparing catalysts, the XRD peaks of metallic Cu^0^ become weaker, whereas the XRD peaks of CuO are enhanced. This reveals that, during the discharge process in the presence of air, metallic Cu^0^ could be oxidized into CuO. For Cu/C-0, Cu/C-10, Cu/C-20, Cu/C-30, Cu/C-60, and Cu/C-120, the XRD peaks of Cu_2_O are similar. Therefore, the amount of Cu_2_O on all of the catalysts could be close.

The full survey XPS spectra confirm the presence of Cu, O and C on Cu/C-0, Cu/C-10, Cu/C-20, Cu/C-30, Cu/C-60, and Cu/C-120 (Supplementary Figure S10). Figure 3B shows the Cu 2p XPS spectra of Cu/C-0, Cu/C-10, Cu/C-20, Cu/C-30, Cu/C-60, and Cu/C-120. Two peaks are present at 932.77 and 952.87 eV on the Cu 2p XPS spectrum of Cu/C-0, and caused by Cu^0^ 2p_3/2_ and Cu^0^ 2p_1/2_, respectively (Ling et al., 2018; An et al., 2019). No evident XPS peaks characteristic of Cu_2_O and CuO can be seen on Cu/C-0. On the Cu 2p XPS spectra of Cu/C-10 and Cu/C-20, peaks at 932.77, 935.50, 941.52, 944.18, 952.87, 955.35, and 962.46 eV appear. The peaks at 932.77, 935.50, 952.87, and 955.35 eV are attributed to Cu^0^ 2p_3/2_, Cu^2+^ 2p_3/2_, Cu^0^ 2p_1/2,_ and Cu^2+^ 2p_1/2_, respectively, while the peaks at 941.52, 944.18, and 962.46 eV are the satellite peaks of Cu^2+^ (Biesinger, 2017; Wu et al., 2018; An et al., 2019; Verma et al., 2022). No evident XPS peaks characteristic of Cu_2_O can be seen on Cu/C-10 and Cu/C-20. On the Cu 2p XPS spectra of Cu/C-30, Cu/C-60, and Cu/C-120, in addition to the peaks of Cu^2+^ and satellite peaks of Cu^2+^, the peaks caused by Cu^+^ are also present at 933.41 and 953.22 eV, and there is no evident peak characteristic of metallic Cu^0^ (Biesinger, 2017; Wu et al., 2018; An et al., 2019; Verma et al., 2022). Based on Cu 2p XPS spectra, the Cu^+^/Cu^2+^ ratios on the surfaces of Cu/C-10, Cu/C-20, Cu/C-30, Cu/C-60, and Cu/C-120 are calculated to be 0.00, 0.00, 0.21, 0.46, and 0.59, respectively.

**FIGURE 4:**
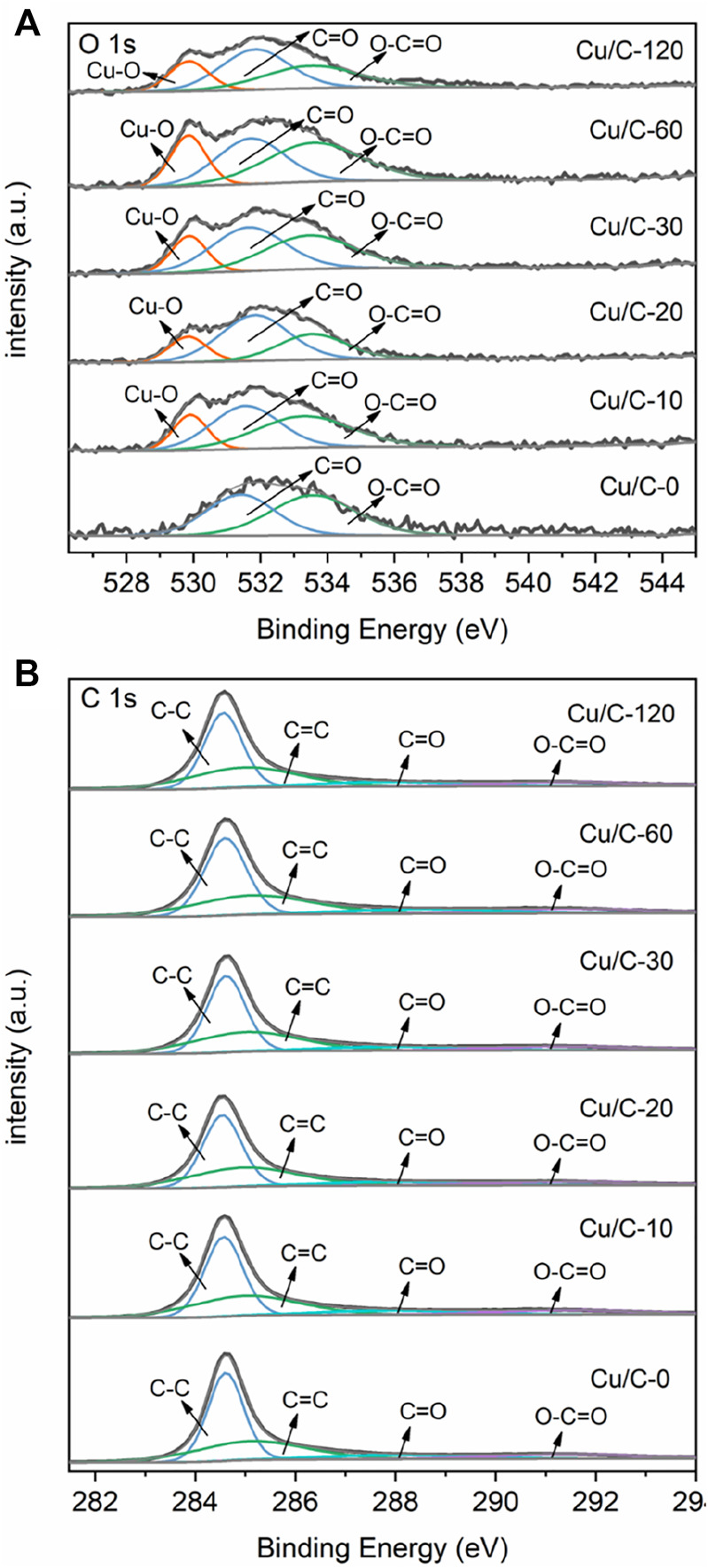
**(A)** O 1s XPS spectra and **(B)** C 1s XPS spectra of Cu/C-0, Cu/C-10, Cu/C-20, Cu/C-30, Cu/C-60 and Cu/C-120.

Figure 4A shows the O 1s XPS spectra of catalysts. On the O 1s XPS spectrum of Cu/C-0, two peaks at 531.41 and 533.57 eV are present, and attributed to the C=O and O-C=O bonds of the carbonyl (C=O) or/and carboxyl (COOH) groups on CNTs, respectively (Men et al., 2022a; Zhao et al., 2022). No evident O 1s XPS peaks characteristic of Cu_2_O and CuO can be seen on Cu/C-0. This is in consistent with the Cu 2p XPS spectrum. On the XPS spectra of Cu/C-10, Cu/C-20, Cu/C-30, Cu/C-60, and Cu/C-120, in addition to the peaks attributed to the C=O or/and COOH groups on CNTs, a Cu-O peak also appears, indicating the presence of copper oxides on Cu/C-10, Cu/C-20, Cu/C-30, Cu/C-60, and Cu/C-120 (Biesinger, 2017; Ling et al., 2018; Wu et al., 2018; An et al., 2019; Verma et al., 2022). As reflected in Figure 4A, along with elongating the discharge time for preparing catalyst, the XPS peak due to Cu-O bond shifts slightly to lower binding energies. This could be caused by the changes in the ratio between Cu_2_O and CuO on the catalysts. Figure 4B illustrates the C 1s spectra of Cu/C-0, Cu/C-10, Cu/C-20, Cu/C-30, Cu/C-60, and Cu/C-120. As reflected in the figure, the C 1s spectra of Cu/C-0, Cu/C-10, Cu/C-20, Cu/C-30, Cu/C-60, and Cu/C-120 are almost the same, and exhibit the peaks characteristic of CNTs. This implies that the discharge process used for preparing catalysts could not influence the structure of CNTs.

**FIGURE 5:**
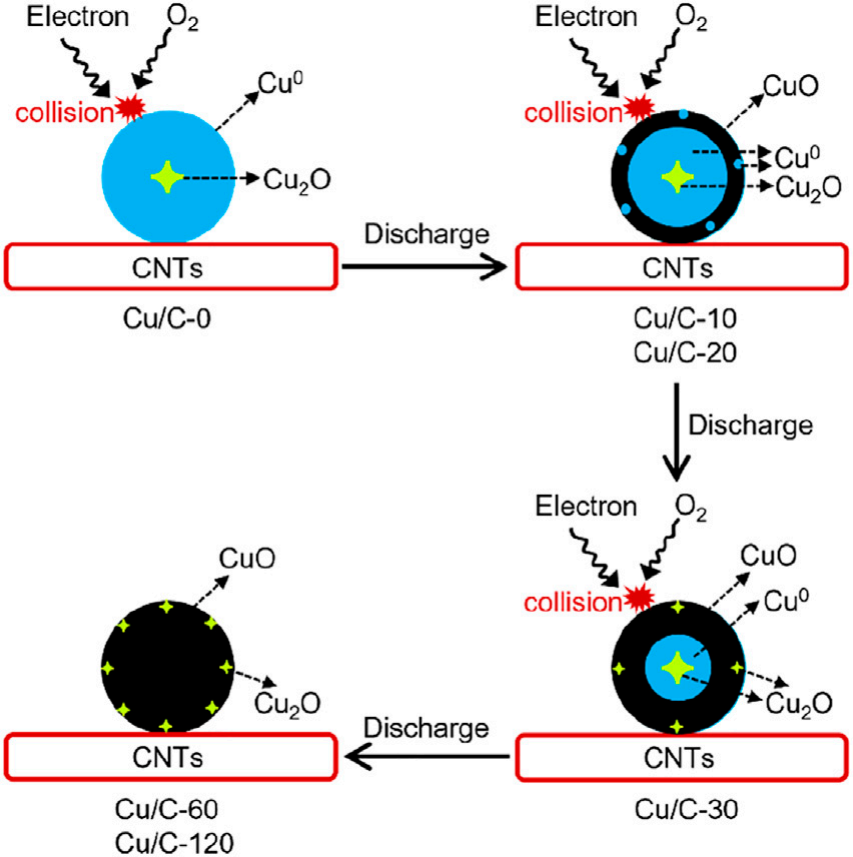
Schematic of the preparation process for the catalysts by using the discharge process.

As reflected by Cu 2p XPS spectra, Cu/C-0 is mainly composed of metallic Cu^0^, while both metallic Cu^0^ and CuO appear on Cu/C-10 and Cu/C-20. As shown by XRD patterns, there are a small amount of Cu_2_O on Cu/C-0, Cu/C-10, and Cu/C-20. However, no evident Cu_2_O peaks can be seen on the Cu 2p XPS spectra of Cu/C-0, Cu/C-10, and Cu/C-20. XPS is an excellent technique to analyze the surface properties of materials, but the detection depth of XPS is smaller than that of XRD. The fact that Cu_2_O is observed on XRD patterns but disappears on XPS spectra indicates that, on Cu/C-0, Cu/C-10, and Cu/C-20, Cu_2_O could be inside of the copper species nanoparticles (Figure 5). During the discharge process, abundant electrons are produced from the ionization of the gas used from triggering the discharge (e.g., Air, O_2_, and Ar) (Sabat et al., 2016; Dou et al., 2018; Men et al., 2020; Men et al., 2022a; Men et al., 2022b). The discharge-produced electrons move fast and have energies as high as 5–10 eV (Sabat et al., 2016; Dou et al., 2018; Men et al., 2020; Men et al., 2022a; Men et al., 2022b). When conducting the discharge process on Cu/C-0 for 10 and 20 min, fierce collisions of the discharge-produced electrons with O_2_ and copper species nanoparticles lead to the oxidation of metallic Cu^0^ into CuO on Cu/C-10 and Cu/C-20 (Figure 5). The amount of CuO produced from conducting the discharge process for 10 and 20 min could be small, and the CuO layer on the surface of the copper species nanoparticles on Cu/C-10 and Cu/C-20 could be thin (Figure 5). In addition, there could be some metallic Cu^0^ on the surface of the copper species nanoparticles on Cu/C-10 and Cu/C-20 (Figure 5). Therefore, the XPS peaks of metallic Cu^0^ can still be observed on Cu/C-10 and Cu/C-20. When the discharge process is elongated to 30 min, more metallic Cu^0^ are oxidized into CuO, and thereby the CuO layer on the surface of the copper species nanoparticles gets thicker (Figure 5). The thicker CuO layer results in the disappearance of the peaks of metallic Cu^0^ on the XPS spectra of Cu/C-30 (Figure 3B), although small peaks of metallic Cu^0^ are observed on the XRD pattern of Cu/C-30 (Figure 3A).

**FIGURE 6:**
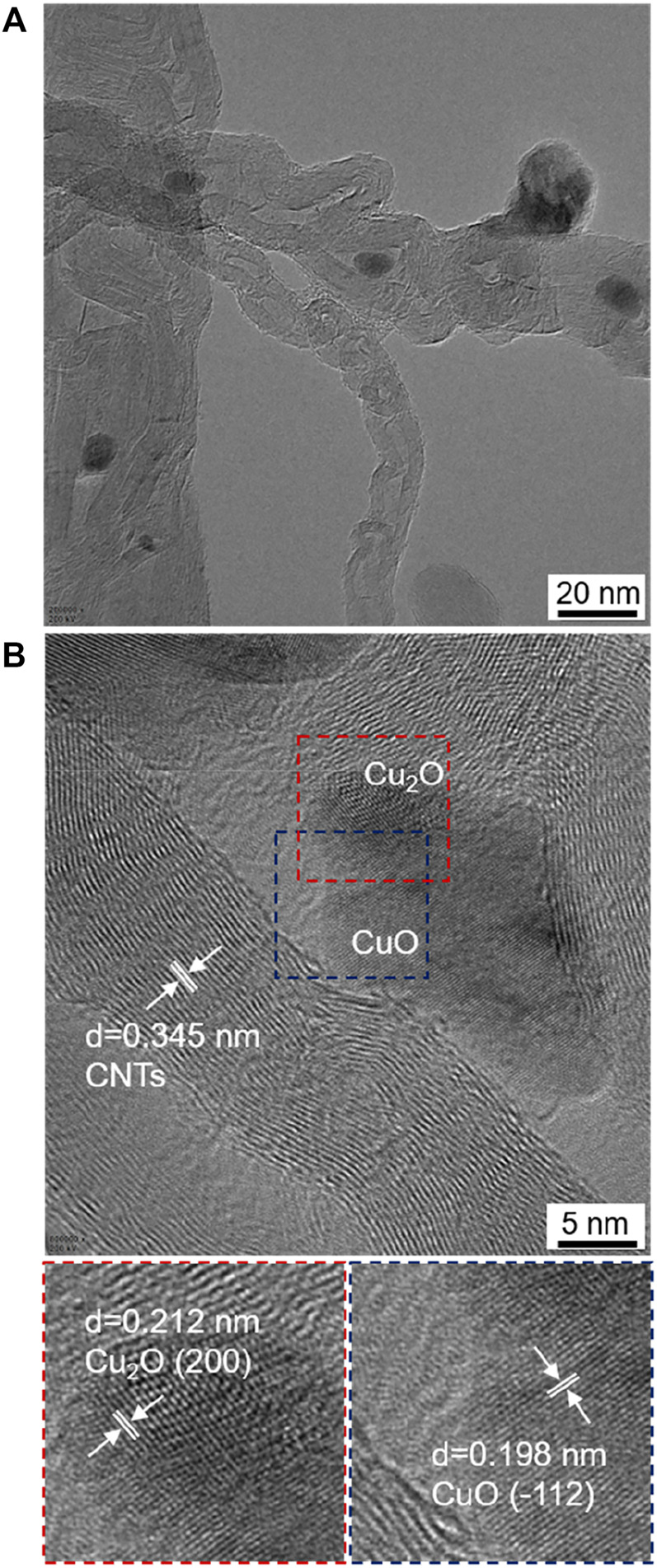
**(A)** TEM image and **(B)** HRTEM images of Cu/C-60.

It has been revealed that, when treating metal oxides by using discharge process, oxygen vacancies can be easily formed on the surfaces of metal oxides, due to the fierce collisions of the discharge-produced electrons with the surfaces of metal oxides (Sabat et al., 2016; Dou et al., 2018; Men et al., 2020; Men et al., 2022a; Men et al., 2022b). Therefore, when conducting the discharge process for 30, 60, and 120 min, fierce collisions of the discharge-produced electrons with the surface of CuO on the catalysts could create oxygen vacancies on the surface of CuO, thus converting some CuO into Cu_2_O (Figure 5) (Sabat et al., 2016; Dou et al., 2018; Men et al., 2020; Men et al., 2022a; Men et al., 2022b). This could be the origin for the presence of Cu^+^ peaks on the XPS spectra of Cu/C-30, Cu/C-60, and Cu/C-120 (Figure 3B). On Cu/C-60 and Cu/C-120, the copper species could mainly be CuO, with some Cu_2_O on the surface of CuO nanoparticles, thus creating Cu_2_O-CuO interface on the catalyst surface. Formation of Cu_2_O-CuO interface on Cu/C-60 is further demonstrated by TEM observations. As shown by TEM and HRTEM images of Cu/C-60 in Figure 6, lattice fringes attributed to Cu_2_O and CuO appear on the nanoparticles supported on CNTs, and Cu_2_O-CuO interface can be clearly seen on the supported nanoparticles.

**FIGURE 7:**
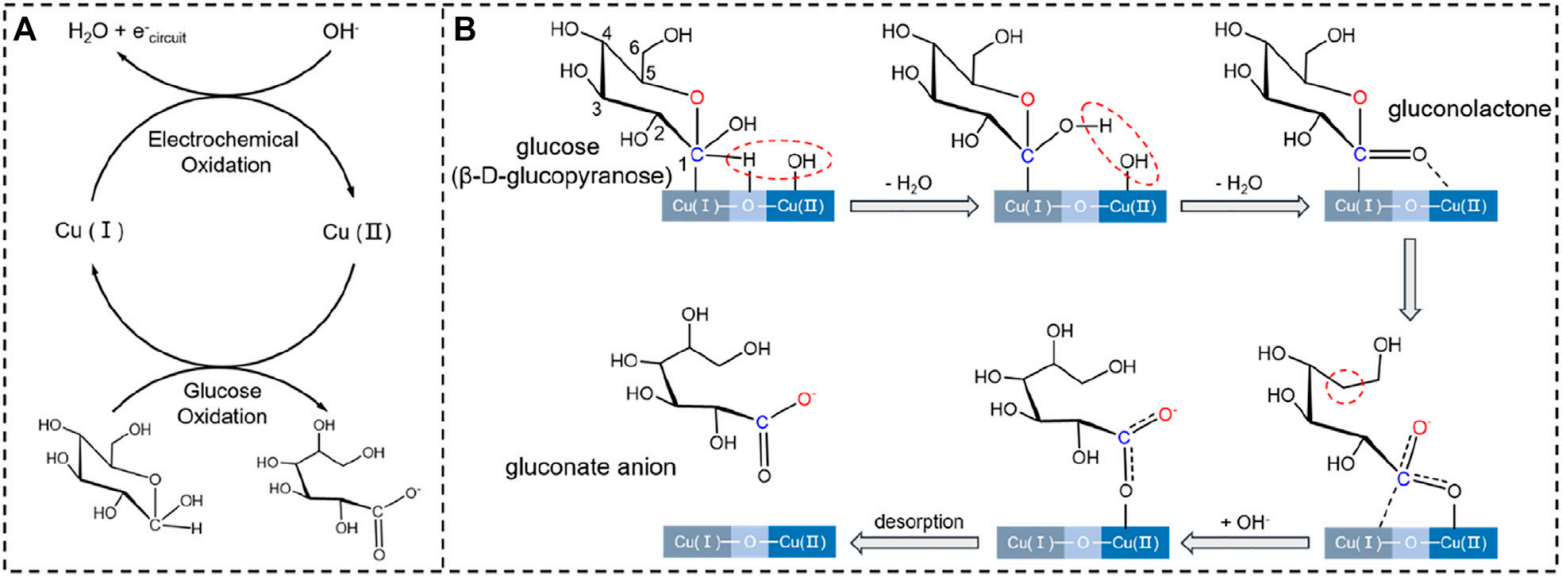
**(A)** Schematic for the redox cycles of Cu^+^/Cu^2+^ and glucose/gluconate in the presence of OH. **(B)** Possible intermediates formed during GOR on Cu/C-60.

The GOR performance of metallic Cu^0^ has been reported to be poorer than those of copper oxides (Biesinger, 2017; Wu et al., 2018; An et al., 2019; Verma et al., 2022). Moreover, the GOR performance of the mixture of copper oxides with copper in different valences, e.g., Cu_2_O/CuO mixture, is better than those obtained by using the copper oxides separately (Biesinger, 2017; Wu et al., 2018; An et al., 2019; Verma et al., 2022). The interfaces among copper oxides with copper in different valences, e.g*.*, Cu_2_O-CuO interface, have been revealed to provide the catalytic active sites for enhancing GOR (Biesinger, 2017; Wu et al., 2018; An et al., 2019; Verma et al., 2022). At the interface, the copper with a lower valence is more favorable for glucose adsorption. Adsorption of glucose on catalyst, e.g., metal oxide, proceeds with electron transfer from catalyst to glucose (Men et al., 2022a). The metal site with a lower valence has a higher electron-donating ability than the metal site with a higher valence, and are thereby more favorable for glucose adsorption (Figure 7) (Men et al., 2022a). In glucose molecule, C_1_ atom bonding to two O atoms has more vacant orbitals to accept the electrons from catalyst (Figure 7) (Men et al., 2022a). Adsorption of glucose on catalyst has been shown to prefer to proceed through the bonding interaction of C_1_ atom of glucose with catalyst. OH groups are indispensable for GOR. Different from glucose adsorption, OH prefers to adsorb at the metal site with a higher valence at the interface on catalyst (Figure 7). Adsorption of OH on catalyst proceeds with electron transfer from OH to catalyst (Men et al., 2022a). The metal site with a higher valence has more vacant orbitals to accept the electrons from OH, and are thereby more flexible for OH adsorption (Men et al., 2022a).

As indicated by XPS and XRD studies, Cu/C-0 is mainly composed of metallic Cu^0^. This could be the origin for the poor GOR performance of Cu/C-0. CuO is present on Cu/C-10, and Cu/C-20, but some metallic Cu^0^ could exist on the surface of Cu/C-10 and Cu/C-20. Therefore, the GOR performances of Cu/C-10 and Cu/C-20 are better than that of Cu/C-0, but still lower than those obtained in the presence copper oxide mixture (Figure 2). Along with the disappearance of metallic Cu^0^ and the presence of Cu_2_O on catalyst surface, the GOR performance is enhanced on Cu/C-30, and reaches to the maximum on Cu/C-60. However, more Cu_2_O are formed on the surface of Cu/C-120. This increases the sites for glucose adsorption, but decreases the sites for OH adsorption, thus making the GOR efficiency of Cu/C-120 lower than Cu/C-60. The Cu_2_O:CuO ratio on Cu/C-60 could be the most favorable for enhancing the adsorption and conversion of both glucose and OH, thus improving the GOR performance.

**FIGURE 8:**
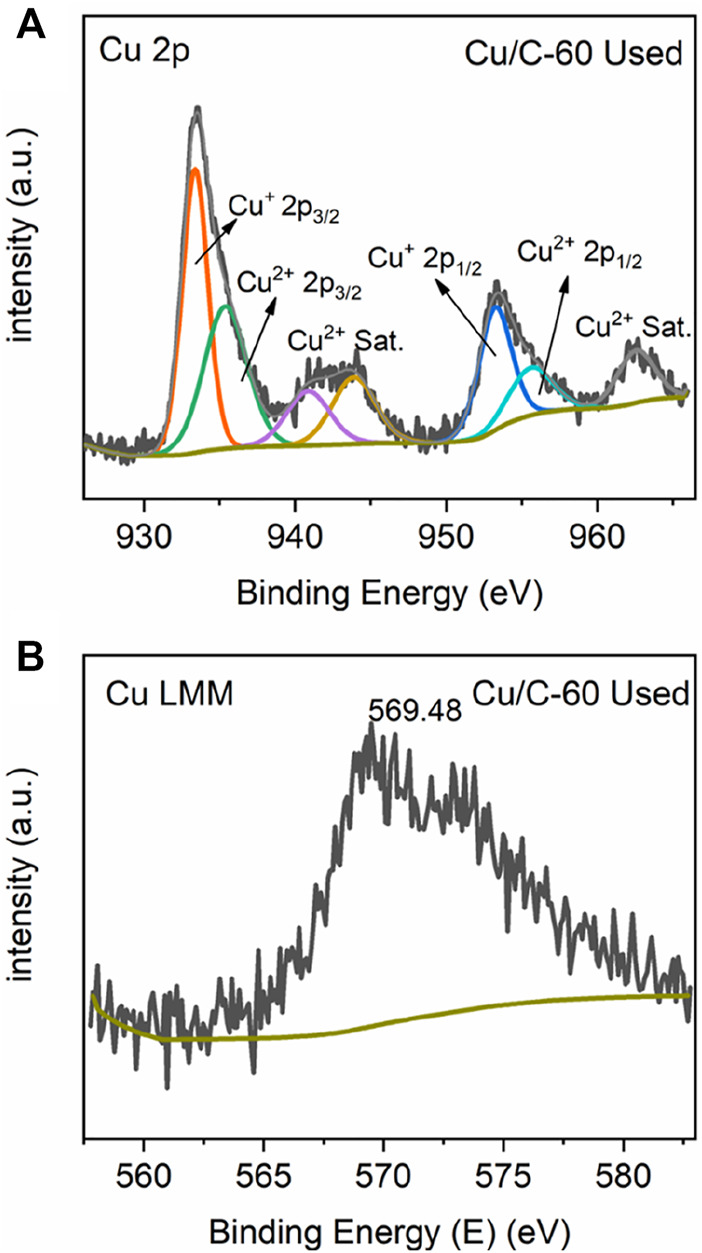
**(A)** Cu 2p and **(B)** Cu LMM XPS spectra of the used Cu/C-60 after GOR reaction.

Figure 7 schematically shows the reaction mechanism of GOR on Cu/C-60. It has been demonstrated detailedly in our previous work that the product of GOR is gluconate anion, and the oxidation of glucose into gluconate anion proceeds in the presence of OH groups mainly *via* five intermediates (Men et al., 2022a). Co-adsorption of glucose and OH at the Cu_2_O-CuO interface on Cu/C-60 leads to the first intermediate. In the first intermediate, C_1_ of glucose bonds to the Cu^+^ site, H atom connecting to C_1_ interacts with the O atom of Cu^+^-O-Cu^2+^ bridge site, and OH group binds to the Cu^2+^ site. In the first step of GOR, the H atom connecting to C_1_ is abstracted by the OH group adsorbed at Cu^2+^, leading to the formation of a H_2_O molecule and the second intermediate. In the second step of GOR, another OH group adsorbed at Cu^2+^ captures the H of the OH group bonding to C_1_ atom, converting the C_1_-O bond into a C_1_ = O bond in the third intermediate (gluconolactone) (Figure 7). In the fourth step of GOR, a C-O bond in the third intermediate is split, resulting in the fourth intermediate. The fourth intermediate bonds to an OH group to form an adsorbed gluconate anion (the fifth intermediate) (Figure 7). After desorption of gluconate, the Cu^+^-O-Cu^2+^ bridge site is recovered for further reactions. We conduct XPS analyses on the used Cu/C-60 after reaction. The Cu 2p XPS spectrum of the used Cu/C-60 after reaction (Figure 8A) is almost the same as that of the fresh Cu/C-60 before reaction (Figure 3B). In the Cu LMM Auger spectrum, the peak at 569.48 eV confirmed the presence of Cu^+^ (Figure 8B) (Verma et al., 2022). The Cu^+^/Cu^2+^ ratio is about 0.42, which is close to that of fresh Cu/C-60 before reaction (0.46). The stable Cu^+^/Cu^2+^ ratio could be the origin for the outstanding stability of Cu/C-60 in GOR.

In addition to the Cu^+^-Cu^2+^ synergetic effect, the CNTs support could also make great contributions to the higher performance of Cu/C-60 in GOR. The CNTs support suppresses the aggregation of the catalytic active Cu_2_O-CuO nanoparticles. As reflected by the TEM image in Figure 6, the catalytic active Cu_2_O-CuO nanoparticles are uniformly distributed on the carbon nanotubes (CNTs) support, and the interface between Cu_2_O-CuO and CNTs is clear. This promotes the interaction of glucose (reactant) with the catalytic active Cu_2_O-CuO nanoparticles, thus improving the reaction efficiency. In addition, the CNTs support is highly conductive, and is thereby flexible for charge transfer. This also helps to enhance the adsorption and conversion of glucose (reactant), thus increasing the reaction efficiency.

### Portable and compact electrochemical system with a three-electrode chip

**FIGURE 9:**
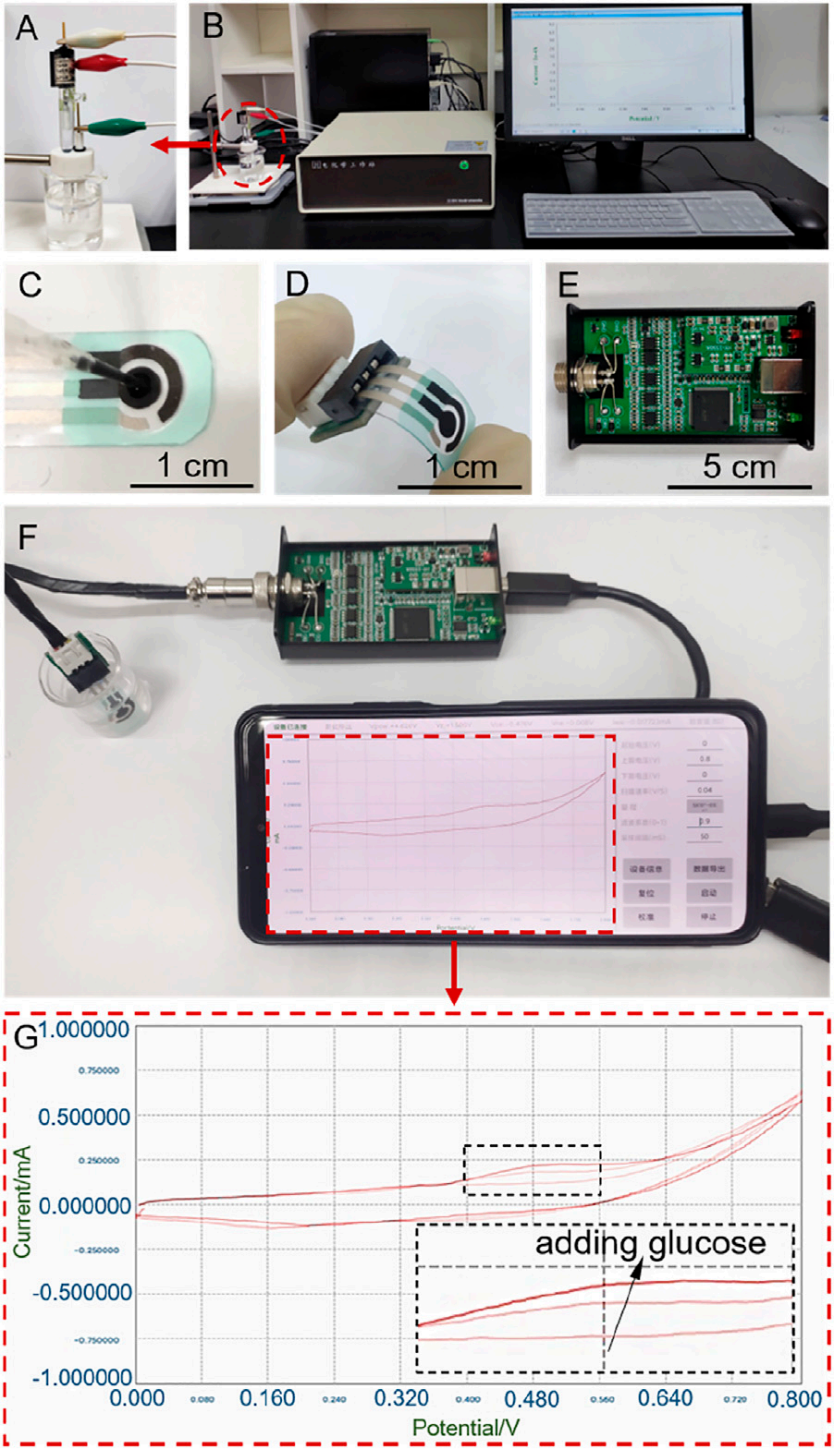
**(A,B)** The bloated electrochemical workstation connecting to a three-electrode system including a working electrode with catalyst, a Pt plate electrode and a saturated calomel electrode. **(C,D)** Image of the three-electrode chip. **(E)** Image of the integrated circuit board. **(F)** Portable and compact electrochemical system including the three-electrode chip, integrated circuit board and mobile phone for recording and displaying the data. **(G)** CV curves recorded on the electrochemical system shown in (**F**).

As discussed above, Cu/C-60 exhibits a better GOR performance than Cu/C-0 and other discharge-prepared catalysts, as shown in Figures 1, 2. However, the GOR performance shown in Figures 1, 2 are obtained on a bloated electrochemical workstation (CHI760E, CH Instrument, Inc.) connecting to a three-electrode system including a working electrode with catalyst (diameter: 0.30 cm), a Pt plate electrode (0.50 cm × 0.50 cm) as a counter electrode and a saturated calomel electrode (SCE) as a reference electrode (Figure 9). If we would like to apply Cu/C-60 to fabricate sophisticated portable device for detecting glucose and realize the popular applications of the device, the electrochemical system for GOR has to be miniaturized, moreover, the data from the electrochemical system had better to be read by mobile phone. To this purpose, we fabricate a portable and compact electrochemical system including a three-electrode chip, integrated circuit board and mobile phone for recording and displaying the data (Figures 9C–G). The three-electrode chip is composed of a polyethylene terephthalate substrate, a conductive silver coating, an insulating layer and three electrodes.

**FIGURE 10:**
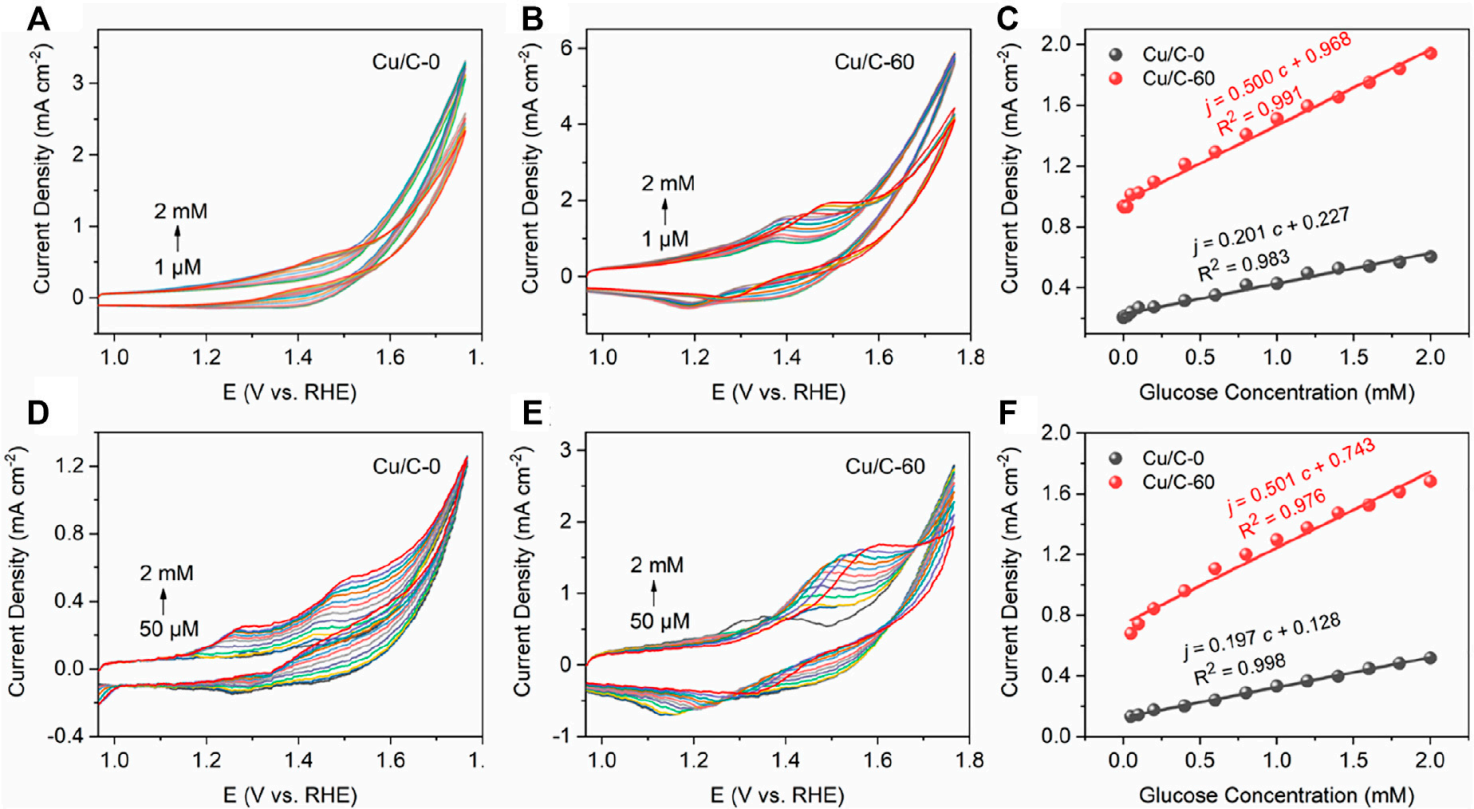
**(A,B)** CV curves recorded by connecting the three-electrode chip (Figure 9) to CHI760E electrochemical workstation which is the same as that used for measuring the data shown in Figures 1, 2. (**C**) Current densities as function of glucose concentrations. The current densities in **(C)** are the peak current densities of the oxidation peaks on the CV curves in **(A,B)**. **(D,E)** CV curves recorded by using the portable and compact electrochemical system (Figure 9). **(F)** Current densities as function of glucose concentrations. The current densities in **(F)** are the peak current densities of the oxidation peaks on the CV curves in **(D,E)**. The CV curves are recorded by adding various concentrations of glucose into the 0.1 M NaOH solution at the scan rate of 40 mV s^−1^.

To test the performance of the three-electrode chip in GOR, we firstly connect the three-electrode chip to the bloated CHI760E electrochemical workstation which is the same as that for measuring the data in Figures 1, 2. Figures 10A,B illustrate the CV curves obtained on the CHI760E electrochemical workstation for Cu/C-0 and Cu/C-60, respectively. Figure 10C shows the calibrated lines of current densities as function of glucose concentrations, with the current densities being the peak current densities of the oxidation peaks on the CV curves shown in Figures 10A,B. The slope of the calibrated line in Figure 10C is the sensitivity of the catalyst loaded on the small three-electrode chip to GOR. As shown in Figure 10C, the sensitivities of Cu/C-0 and Cu/C-60 loaded on the three-electrode chip to GOR are 201 and 500 μA mM^−1^ cm^−2^, respectively (Figure 10C). These are close to those shown in Figure 2. In addition, the detection limits for Cu/C-0 and Cu/C-60 loaded on the small three-electrode chip are both 1 μM (Figures 10A,B).

To further simplify the electrochemical system, we connect the three-electrode chip to an integrated circuit board and mobile phone to form a portable and compact electrochemical system. Figures 10D,E illustrates the CV curves obtained on the portable and compact electrochemical system. Figure 10F shows the calibrated lines of current densities as function of glucose concentrations, with the current densities being the peak current densities of the oxidation peaks on the CV curves shown in Figures 10D,E. The slope of the calibrated line in Figure 10F is the sensitivity of the catalyst loaded on the small three-electrode chip to GOR. As shown in Figure 10F, the sensitivities of Cu/C-0 and Cu/C-60 loaded on the small three-electrode chip to GOR are 197 and 501 μA mM^−1^ cm^−2^, respectively (Figure 10C). These are close to those obtained on the CHI760E electrochemical workstation. This indicates the accuracy, reasonability and applicability of our portable and compact electrochemical system. In addition, the detection limits for Cu/C-0 and Cu/C-60 in the portable and compact electrochemical system are both 50 μM (Figures 10D,E). This is higher than those obtained by using the bloated CHI760E electrochemical workstation, but is much lower than the common blood glucose concentration of human body (>3 mM) (Sud et al., 2015). Therefore, our portable and compact electrochemical system is excellent for detecting trace amount of glucose.

## Conclusion

In summary, a noble-metal-free Cu/C-60 catalyst is fabricated by supporting Cu_2_O-CuO nanoparticles on CNTs through a novel discharge process. In GOR process, Cu/C-60 exhibits a sensitivity as high as 532 μA mM^−1^ cm^−2^, a detection limit as low as 1 μM and a steady-state response time of only 5.5 s. Moreover, Cu/C-60 has outstanding stability and anti-interference ability to impurities. The synergistic effect of Cu_2_O-CuO as well as the CNTs support could improve the adsorption and conversion of glucose and OH groups, thus enhancing GOR performance. With Cu/C-60 as the GOR catalyst, we fabricate a three-electrode chip. A portable and compact electrochemical system is constructed by connecting the three-electrode chip with Cu/C-60 to an integrated circuit board and a mobile phone for recording and displaying data. The portable and compact electrochemical system results in a GOR sensitivity of 501 μA mM^−1^ cm^−2^, which is close to the data measured on the bloated electrochemical workstation. The detection limit of the portable and compact electrochemical system in GOR is 50 μM. This is higher than those obtained on the bloated electrochemical workstation, but is much lower than the common blood glucose concentration of human body (>3 mM). This indicates the accuracy, reasonability and applicability of the portable and compact electrochemical system. These results are helpful for fabricating fast, efficient and portable devices for detecting trace amount of glucose in blood and food.

## Data Availability

The original contributions presented in the study are included in the article/Supplementary Material, further inquiries can be directed to the corresponding authors.
