# Supplementary material for: Fast and efficient electrocatalytic oxidation of glucose triggered by Cu2O-CuO nanoparticles supported on carbon nanotubes
Source: Front Chem. 2022 Sep 1;10:998812. doi: 10.3389/fchem.2022.998812 (PMC9475138; doi:10.3389/fchem.2022.998812)
Supplement: Supplementary file 1 [file DataSheet1.docx]

Supplementary Material

**Fast and Efficient Electrocatalytic Oxidation of Glucose Triggered by Cu_2_O-CuO Nanoparticles Supported on Carbon Nanotubes**

**Zhongting Wang^1^, Yi Liu^2^,** **Yongxi Cheng^3^, Yu-Long Men^1^, Peng Liu^1^, Lei Zhang^4*^, Bin Dai^1*^ and Yun-Xiang Pan^1, 2*^**

^1^ *School of Electronic Information and Electrical Engineering, Shanghai Jiao Tong University, Shanghai 200240, P. R. China*

^2^ *Department of Chemical Engineering, School of Chemistry and Chemical Engineering, Shanghai Jiao Tong University, Shanghai, 200240, P. R. China*

^3^ *Beijing Institute of Aerospace Testing Technology, Beijing, 100074, P. R. China*

^4^ *Department of Vascular Surgery, Changhai Hospital, Naval Medical University, Shanghai, 200433, P. R. China*

*** Corresponding Authors**

Bin Dai (E-mail: daibin@sjtu.edu.cn)

Lei Zhang (E-mail: heatstones@yeah.net)

Yun-Xiang Pan (E-mail: yxpan81@sjtu.edu.cn)


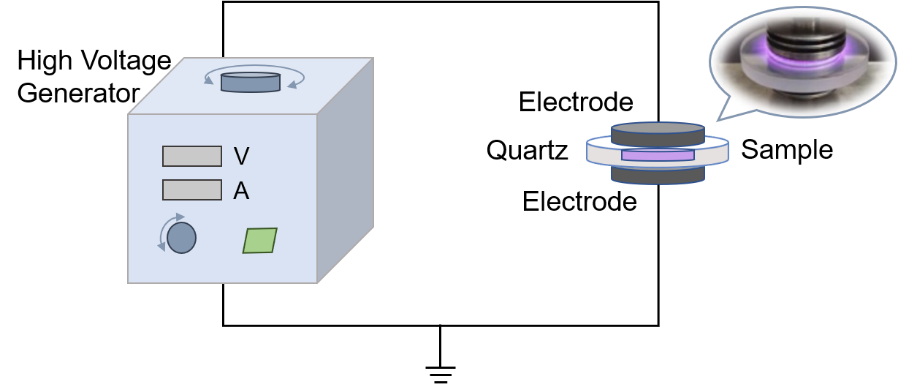


**Supplementary Figure S1.** Schematic for the discharge set-up.





**Supplementary Figure S2.** Current density as a function of time on Cu/C-60 at different potentials (*vs.* SCE).





**Supplementary Figure S3.** GOR sensitivity of Cu/C-10, Cu/C-20, Cu/C-30, Cu/C-60 and Cu/C-120.


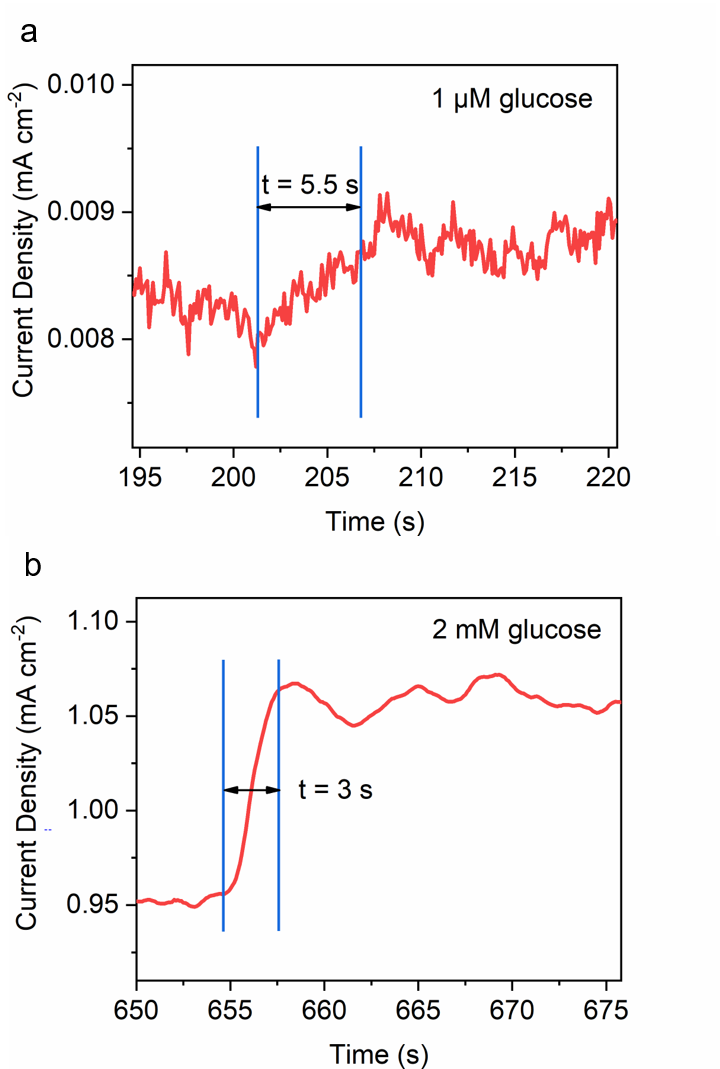


**Supplementary Figure S4.** Response times of Cu/C-60 at the glucose concentration of (a) 1 μM and (b) 2 mM.





**Supplementary Figure S5**. Stability of Cu/C-60 in GOR.





**Supplementary Figure S6**. Anti-interference ability of Cu/C-60 to impurities in GOR.


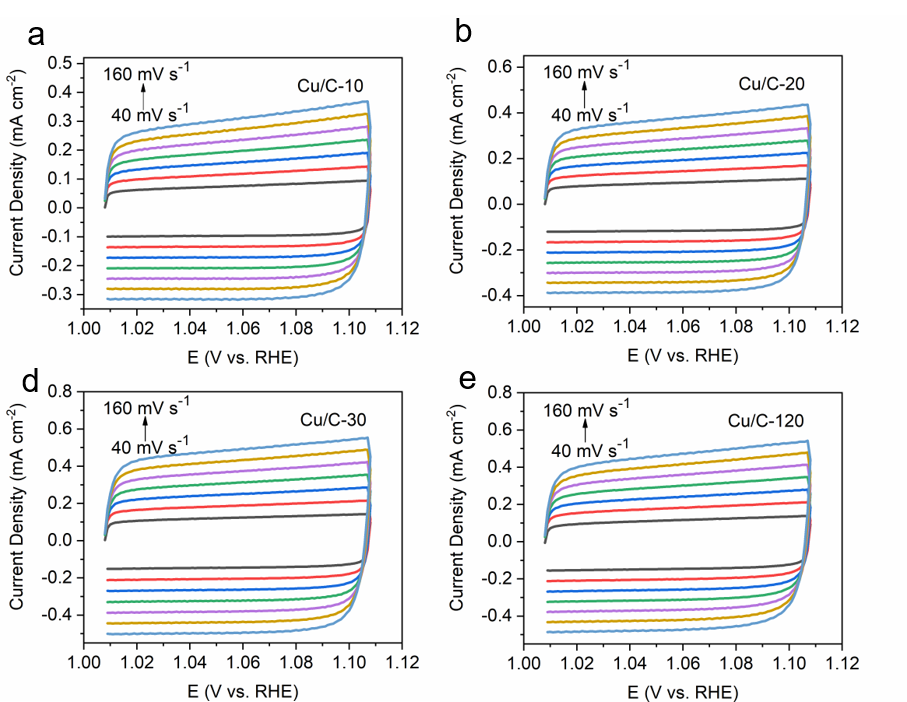


**Supplementary Figure S7.** CV curves in the non-Faradaic region for (a) Cu/C-10, (b) Cu/C-30, (c) Cu/C-60 and (d) Cu/C-120 at scan rates ranging from 40 to 160 mV s^-1^.





**Supplementary Figure S8.** *C_dl_* of Cu/C-10, Cu/C-20, Cu/C-30, Cu/C-60 and Cu/C-120.


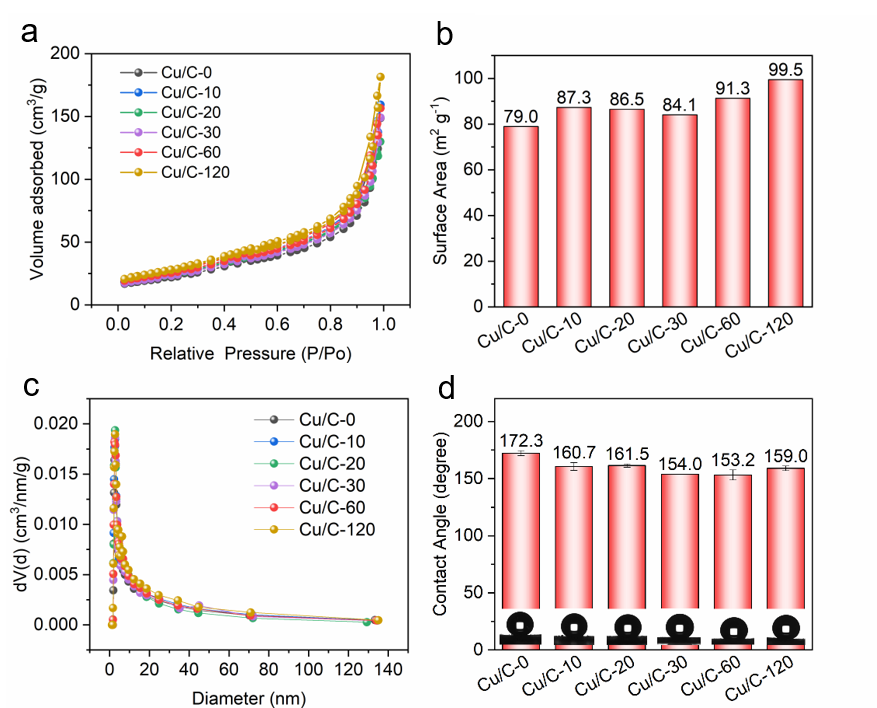


**Supplementary Figure S9.** (a) Nitrogen adsorption-desorption isotherm, (b) surface area, (c) pore size distribution and (d) contact angles for Cu/C-0, Cu/C-10, Cu/C-20, Cu/C-30, Cu/C-60, and Cu/C-120.





**Supplementary Figure S10.** Full survey XPS spectra of Cu/C-10, Cu/C-20, Cu/C-30, Cu/C-60 and Cu/C-120.
